# Suspected Symptomatic Infected Native Aortic Aneurysm Turns Out To Be Aortic Tumour Originating From Metastatic Cancer of Unknown Primary: A Case Report and Review of Literature

**DOI:** 10.1016/j.ejvsvf.2024.06.002

**Published:** 2024-07-04

**Authors:** Laina Passos, Christian Zielasek, Drosos Kotelis, Vladimir Makaloski, Michel Bosiers

**Affiliations:** Department of Vascular Surgery, University Hospital Bern, University of Bern, Bern, Switzerland

**Keywords:** Aortic tumour, Cancer of unknown primary, Open aortic repair, Y graft vascular prosthesis

## Abstract

**Introduction:**

The non-specific clinical presentation of a primary aortic tumour may mimic infectious processes. Together with its rarity, this resemblance can complicate timely identification and pose diagnostic challenges.

**Report:**

The case of a 77 year old male patient complaining of abdominal pain radiating to the back, fatigue, and loss of appetite for a month, is presented. Contrast enhanced computed tomography showed a 47 mm infrarenal aortic aneurysm with peripheral enhancement. With suspicion of an infected native aortic aneurysm, open aortic repair was performed using a bovine pericardial Y prosthesis. The intra-operative biopsy revealed a malignant undifferentiated neoplasm, which later turned out to originate from metastatic cancer of unknown primary. The patient died six months later following comprehensive and extensive oncological treatment, which included radiotherapy and chemotherapy.

**Discussion:**

Given the scarcity of literature and challenges in classification, treatment recommendations rely on a multidisciplinary approach, involving surgery, radiotherapy, and chemotherapy. Despite the lack of established guidelines, early intervention, even in metastatic cases, may improve clinical outcomes. Surgical resection, whenever appropriate, is advocated, as it not only alleviates symptoms, but intra-operative histological sampling also aids in obtaining a definitive diagnosis.

## Introduction

Vascular tumours include a large and heterogeneous spectrum of lesions.^1^^,^^2^ The diagnostic process is inherently challenging due to various non-specific clinical manifestations, the frequent rapid dissemination of the disease, and the rarity of cases, particularly evident in primary vascular tumours like primary aortic tumours.^3^^,^^4^ As a result, early identification and proper treatment of such tumours is often difficult. Here, the case of a symptomatic infrarenal aortic process, which was later identified as a tumour originating from metastatic cancer of unknown primary (CUP) is presented.

## Case report

A 77 year old man complaining of abdominal pain radiating to the back for the previous month, accompanied by fatigue and loss of appetite, without fever was transferred this institution from another hospital. Due to abdominal pain, he consulted his general practitioner one day before being transferred to hospital, where slightly elevated inflammatory markers were observed, and an ultrasound showed enlargement of the aorta. Upon admission, blood cultures were obtained, which subsequently demonstrated no microbial growth. Laboratory analyses demonstrated slightly elevated inflammatory markers, including a C reactive protein of 84 mg/L, alongside normal leucocyte counts. During the initial assessment, the infectious disease team was consulted and opted to defer initiation of antibiotic therapy, reserving it for instances of fever or instability. A pre-operative transthoracic echocardiogram was not performed. He had no cardiovascular risk factors and was taking no regular medication. His medical history included an appendicectomy in adolescence and inguinal hernia surgery a few years prior.

**Figure 1:**
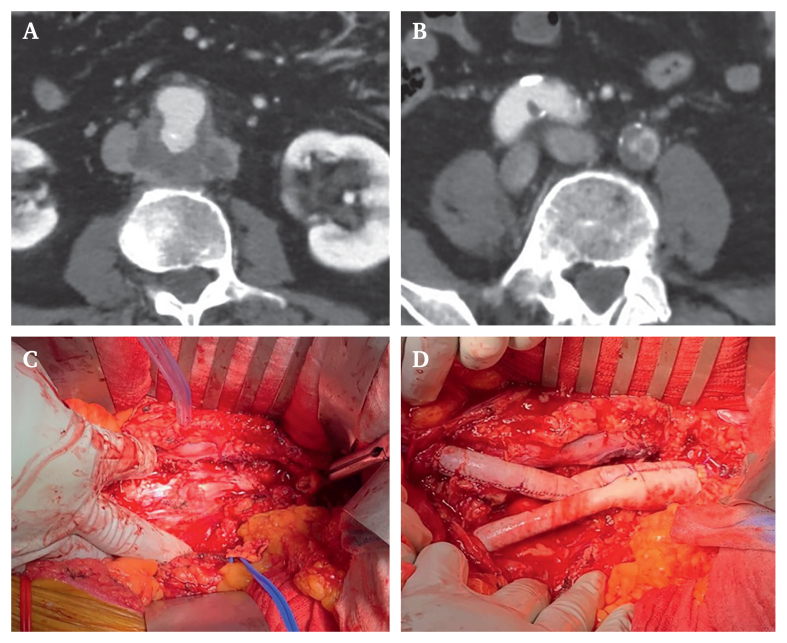
Pre-operative contrast enhanced CT showing (A) the perivascular mass around the infrarenal aorta and (B) the aortic bifurcation. Intra-operative images showing (C) the complete resection of the infrarenal aorta and (D) the bovine reconstruction.

Preoperative contrast enhanced computed tomography (CT) (Fig. 1 and Supplementary Video 1) of the chest and abdomen showed significant atherosclerosis of the abdominal aorta and iliac arteries with evidence of a dorsal ulcer of the infrarenal abdominal aorta, extending into an irregular, peripherally contrast enhancing aneurysm with a maximum diameter of 42 mm × 47 mm. It also revealed a high grade stenosis of the left common iliac artery. There was no supradiaphragmatic focus of infection. With a suspected diagnosis of a symptomatic infected native aortic aneurysm, the need for surgical intervention was indicated. After obtaining informed consent, the patient was promptly taken to the operating room. During surgery, a transoesophageal echocardiogram was performed, which excluded endocarditis and showed a normal pump function.

Supplementary video related to this article can be found at https://doi.org/10.1016/j.ejvsvf.2024.06.002.

The following is/are the supplementary data related to this article:

Video S1 Preoperative contrast-enhanced CT showing perivascular mass around the infrarenal aorta and the aortic bifurcation.1

Video S2 Contrast-enhanced CT three months postoperatively showing significant tumour progression at the level of the infrarenal aorta (left panel) as well as the aortic bifurcation.2

A midline laparotomy was performed to resect the entire infrarenal aorta, including the proximal and middle portions of both common iliac arteries. A large defect was seen in the posterior wall. There was evidence of necrotic tissue, and excessive debridement was performed. No firm mass could be witnessed, and no clear plain for resection. The arterial reconstruction was performed using a physician made bovine pericardial Y graft (Fig. 1). The aortic tissue at the level of the anastomosis seemed healthy. The reconstruction was covered with the greater omentum. During surgery, samples for microbiological and histopathological examination were taken. There was no visual suggestion of a malignant tumour.

**Figure 2:**
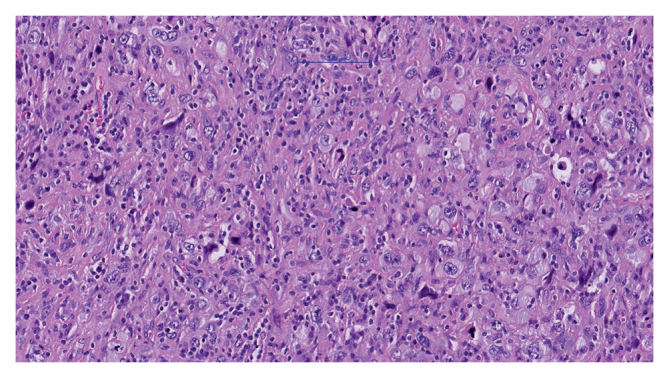
Haematoxylin and eosin stain of undifferentiated pleomorphic sarcoma at 20× magnification showing pleomorphic cells with hyperchromatic nuclei, irregular shapes and mitotic figures.

After surgery, the patient was transferred to the intermediate care unit. Within the following hours, he developed paroxysmal atrial fibrillation, which was treated with amiodarone and metoprolol. The further post-operative course was uneventful with recovery within the expected timeframe. This facilitated the discharge of the patient home in satisfactory overall health. A few days after hospital discharge, the results of the intra-operative biopsy were obtained. Histopathological examination revealed a malignant undifferentiated neoplasm with a high probability of undifferentiated pleomorphic sarcoma (Fig. 2). PD-L1 expression was 25%. This led to the recommendation for a positron emission tomography (PET)-CT scan.

**Figure 3:**
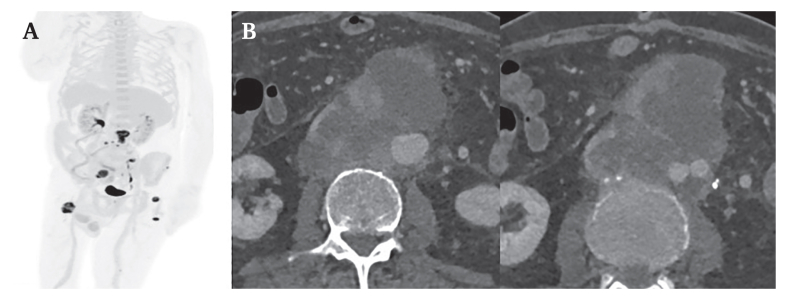
(A) Post-operative positron emission tomography computed tomography (CT) showing a highly metabolically active perivascular mass at the level of the infrarenal aorta with surrounding nodular lesions and bone metastasis. (B) Contrast enhanced CT three months post-operatively showing significant tumour progression at the level of the infrarenal aorta (left panel) as well as the aortic bifurcation.

The PET-CT (Fig. 3A) revealed a highly metabolically active retroperitoneal soft tissue mass with possible infiltration of the inferior vena cava and surrounding nodular lesions. Differential diagnoses included lymph node metastases or satellite lesions and, much less likely, post-operative reactive lymphadenitis. The scan also revealed bone metastases of the left lateral mass of the ilium and the proximal femur on both sides, along with multiple other metastatic soft tissue lesions, with no clearly identifiable primary tumour. Further molecular pathological examination did not provide any clarity regarding the origin of the malignancy, thus confirming the diagnosis of CUP. After careful consideration in a multidisciplinary tumour board, a recommendation for palliative chemotherapy and targeted analgesic radiotherapy was made.

Three months after the initial operation the patient was hospitalised again, due to a deterioration in his general condition. Laboratory analysis showed high levels of C reactive protein (157 mg/L). Blood cultures revealed growth of *Staphylococcus aureus* in one of four samples, prompting initiation of antibiotic therapy. Follow up CT showed no evidence of graft infection. However, there was significant tumour progression in the abdominal aorta, with extensive contact and infiltration around the Y prosthesis (Fig. 3B and Supplementary Video 1). Additionally, there were metastases in the axial and pelvic skeleton, along with several large lymph node metastases exhibiting central necrosis in the mesenteric and left common iliac regions.

The almost complete compression of the inferior vena cava due to the progressing tumour condition presented without symptoms, thus not requiring further treatment apart from rivaroxaban, which the patient was already taking for cardiac dysrhythmia.

A transoesophageal echocardiogram showed thickening of the aortic and mitral valves, without evidence of endocarditis. Due to the very poor prognosis of the oncological condition, the patient was transferred to the palliative care ward where he died six months after the initial surgery and diagnosis.

## Discussion

Malignant aortic tumours are exceptionally rare, with few reported cases. They can be divided into primary and secondary (metastatic) origin.^5^ Primary tumours mainly comprise sarcomas, with angiosarcomas being the most frequent at around 37%. However, with only 1% of all sarcomas exhibiting endothelial differentiation and only a fraction of them occurring in the aorta, even angiosarcomas are extremely rare.^5^^,^^6^ Secondary tumours are slightly more common and mostly originate from pulmonary or oesophageal cancer and thymoma in the thoracic aorta and from retroperitoneal sarcomas and germ cell tumours in the abdominal aorta.^7^ Aortic tumours exhibit a higher prevalence in men, peaking at around the age of 60 and often affecting the abdominal aorta (42%).^5^^,^^8^ No definitive risk factors are identified, although some studies suggest a potential link to Dacron vascular grafts, but this may be coincidental.^8^^,^^9^

The rarity of malignant aortic tumours complicates their classification and treatment. Diagnosis often requires advanced, individualised techniques and is frequently delayed.^10^ In this case, initial CT misdiagnosed the condition, leading to surgery for a suspected aortic infection. The correct diagnosis was achieved post-operatively through histopathological examination and PET-CT.

The clinical signs of malignant aortic tumours are variable, with 30% of patients reporting back pain or weakness and 10% experiencing fever, often mimicking an aortic infection. With an incidence of 15–25%, claudication or peripheral embolisation occurs in 15–25% of cases due to luminal compression or infiltration, as in the present case, and to the thrombogenic potential of tumours in general. Management relies on comprehensive patient assessment and their anatomical features. For the suspected infected native aortic aneurysm, a surgical approach with complete resection was deemed the optimal treatment option. This not only alleviated the symptoms but also provided the final malignancy diagnosis.

Therefore, for suspected or proven malignant aortic tumours, surgical resection to the greatest extent possible with intra-operative collection of microbiological and histological samples is advocated. In terms of graft options, the autologous femoral vein presents a viable alternative, particularly for younger patients with aortic graft infections, despite the associated significant morbidity and mortality risks. Nonetheless, the standard of care in the department remains the use of bovine pericardium. In carefully selected patients presenting with extensive comorbidities, a hostile abdomen, or an overall poor prognosis, an endovascular approach may emerge as a viable option. Given the infrequency of this disease and the limited availability of literature, there are currently no established treatment guidelines. Nevertheless, surgery stands as the primary treatment for resectable tumours. Some studies suggest that non-metastatic patients who undergo surgery generally enjoy a recurrence and survival advantage compared with those who do not undergo resection. Unfortunately, the rate of metastatic disease is very high with 56–85% at the time of diagnosis.^5^

Therefore, a multidisciplinary approach involving oncology evaluation, vascular surgery, and critical care is warranted for an optimal clinical outcome. Unfortunately, due to the challenges associated with the latency in diagnosis and early metastasis, the overall prognosis for these patients is unfavourable, characterised by a median survival of 11 months and a five year overall survival rate of less than 30%.

### Conclusions

A rare case of an infrarenal aortic tumour originating from metastatic CUP is reported. Clinical presentation of malignant aortic tumours is often non-specific and may mimic infective diseases, leading to delays in the correct diagnosis. Due to the extreme rarity of the disease, there are no established treatment guidelines. However, since symptoms such as peripheral embolisation, claudication, or back pain often arise from local infiltration, they may respond to surgical resection, even in the metastatic stage. Surgery should therefore be considered after a complete diagnostic evaluation and in selected cases within a multidisciplinary treatment approach. In cases where no definitive diagnosis can be obtained, histological sampling during open surgery may constitute a crucial diagnostic step.

## Conflict of interest

None.
